# A Poliovirus Receptor (CD155)-Related Risk Signature Predicts the Prognosis of Bladder Cancer

**DOI:** 10.3389/fonc.2021.660273

**Published:** 2021-06-03

**Authors:** Cong Luo, Wenrui Ye, Jiao Hu, Belaydi Othmane, Huihuang Li, Jinbo Chen, Xiongbing Zu

**Affiliations:** ^1^ Department of Urology, Xiangya Hospital, Central South University (CSU), Changsha, China; ^2^ Clinical Medicine Eight-year Program, Xiangya Medical School of Central South University, Changsha, China; ^3^ Department of Neurosurgery, Xiangya Hospital, Central South University (CSU), Changsha, China

**Keywords:** poliovirus receptor, *CD155*, bladder cancer, prognosis, risk signature, nomogram, immune infiltration

## Abstract

**Background:**

Bladder cancer is an aggressive and heterogeneous disease associated with high morbidity and mortality. And poliovirus receptor (*PVR* or *CD155*) played crucial roles in tumor immune microenvironment and cancer development. However, their association remains obscure.

**Methods:**

A total of 797 patients from TCGA and GEO databases were employed in our study, in which 285 cases were set as the training cohort and 512 were defined as the validation cohort. Our own Xiangya cohort with 57 samples was also used for the validation. Survival differences were evaluated by Kaplan-Meier analysis between groups. The immune infiltration was evaluated by ESTIMATE, TIMER, and CIBERSORT algorithms. The risk signature was constructed by LASSO Cox regression analysis. And a nomogram model was generated subsequent to the multivariate Cox proportional hazards analysis to predict 3- and 5-year survival of patients with bladder cancer.

**Results:**

*PVR* was overexpressed across various cancers including bladder cancer and related to poorer overall survival in bladder urothelial carcinoma (BLCA). Samples with higher World Health Organization (WHO) grade or higher tumor stage tended to express higher level of *PVR*. And *PVR*-related genes were involved in several immune processes and oncological pathways. When the patients were divided into low- and high-risk groups based on their risk scores, we found that patients in the high-risk group had shorter overall survival time. Besides, samples with high risk were consistently correlated with tumor hallmarks and higher abundance of immune infiltration. Additionally, chemotherapy showed potent efficacy in high-risk group. Moreover, a nomogram including clinicopathologic features and the established risk signature could predict 3- and 5-year survival in patients with bladder cancer.

**Conclusion:**

Our study revealed that *PVR* was overexpressed and related to poor prognosis in bladder cancer. A risk signature and nomogram model based on *PVR*-related genes could predict the prognosis and therapeutic efficacy and were also associated with the immune infiltration in bladder cancer.

## Introduction

Bladder cancer is the ninth most common cancer in the world, with an estimated 430,000 new diagnoses worldwide per year, resulting in 165,000 deaths annually (1). Approximately a quarter of these cases are muscle invasive bladder cancer (MIBC) (2), ranging from T2 tumors (invading the muscularis propria) to T4 tumors (invading the prostate, uterus, vagina, bowel, or abdominal wall). Unlike non-muscle invasive bladder cancer (NMIBC), these tumors are biologically aggressive, and the 5-year survival rate of untreated patients is < 15%. About 50% of patients die of metastatic diseases despite radical cystectomy with pelvic lymph node dissection, which is the current gold standard treatment for localized T2-T4 MIBC. Efforts to improve outcomes have focused on perioperative chemotherapy to eradicate micro-metastatic disease. However, the prognosis of patients with bladder cancer has not substantially changed over the past 30 years despite the introduction of these therapies (3).

Current advances in the molecular understanding of bladder cancer have led to the identification of promising novel biomarkers and therapeutic targets that can lead to improved outcomes for patients suffering from MIBC on the basis of the intrinsic biology of this tumor type (4). Over the past five years, immune checkpoint inhibitors targeting the programmed cell death protein 1 (PD-1) and its ligand (PD-L1) have demonstrated the ability to relieve immune suppressive phenotype and achieve durable objective responses in trials of patients with metastatic disease, with the promise of changing the therapeutic landscape and improving survival outcomes for patients with this tumor (5). However, a considerable number of patients still acquired drug resistance (6). Therefore, novel immune targets with high safety are in urgent need for the hope to promote response rate and improve patient outcomes when combined with existing immunotherapies.

Cell adhesion molecule poliovirus receptor (PVR, CD155, Necl5, or Tage4) is an appealing target with immunomodulatory functions (7), since it has been shown to mediate oncologic immunity by interacting with receptors on T and natural killer (NK) cells to regulate the function of tumor infiltrating lymphocytes (8). PVR expressed by tumor and tumor-associated myeloid cells can interact with three lymphocyte-expressed receptors to modulate their functions. The inhibitory checkpoint receptors TIGIT (T cell Ig and ITIM domain) and CD96 are upregulated by T and NK cells following activation when exposed chronically to antigen in settings like cancer (9). These receptors compete with the costimulatory receptor DNAX accessory molecule 1 (DNAM1, or CD226) for binding to PVR and function over time to suppress T and NK cell functionality in the tumor microenvironment (TME) (10, 11). The significant immune suppression induced by PVR highlights its potential as a therapeutic target. Previous studies showed that blockade of TIGIT/PVR signaling could reverse T and NK cell dysfunction in various cancers (12). Notably, CD155 on tumor cells was reported to drive resistance to immunotherapy by inducing the degradation of CD226 in CD8+ T cells (13). As such, blocking this interaction with PVR in the TME might be an effective therapy for tumor control.

Overexpression of PVR has been reported in many cancers including melanoma (14), lung adenocarcinoma (15), ovarian cancer (16), myeloid leukemia (17), neuroblastoma (18), pancreatic cancer (19), colorectal cancer (20), hepatocellular carcinoma (21), and bladder cancer (22). The increased *PVR* expression level has been associated with poor clinicopathologic features including lymph node metastasis, a reduction in tumor-infiltrating lymphocytes, tumor histological grade, and poor prognosis in retrospective studies among various solid cancer types (19, 23). To better understand the relationship between *PVR* and clinical correlates, several clinical trials are attempting to explore *PVR* expression in tumor tissue prior, or following the treatment in bladder cancer and other solid cancers (**Supplementary Table 1**).

A study including 228 nonmetastatic muscle-invasive bladder cancer patients collaborated the correlation between high *PVR* expression and shorter survival (22). However, current studies seldom clarify the role of *PVR* in primary bladder cancer. We herein conducted a comprehensive analysis based on several independent cohorts from open databases, plus our own cohort to explore the profile of *PVR* in bladder cancer in order to better understand its biological functions there. Furthermore, a risk signature based on *PVR*-related genes was constructed to predict the BLCA patient prognosis. Additionally, a nomogram model combining the signature and clinicopathologic characteristics was established for higher prediction accuracy.

## Materials and Methods

### Data Extraction

All transcriptome data, genetic mutation data, and clinical characteristics of enrolled samples were extracted from TCGA and GEO databases. A total of 797 primary bladder cancer samples with detailed clinical information were enrolled in our study, in which 285 samples (70%) extracted from the TCGA database (TCGA285) were defined as the training cohort (24); 123 samples (30%) extracted from the TGGA database (TCGA123) were defined as the internal validation cohort, 389 samples extracted from the GEO database [165 in GSE13507 (25, 26), 224 in GSE32894 (27)] were defined as the external validation cohorts. Fragments per kilobase million (FPKM) values were transformed into transcripts per kilobase million (TPM) values, which are more comparable between samples.

### Expression, Survival, and Mutation Analysis of *PVR*

At first, we studied the differential expression of *PVR* between tumor and adjacent normal tissues in TCGA pan-cancer cohorts. *PVR* expression in stratified subgroups was explored in both TCGA BLCA and GSE13507 cohorts. In addition, we obtained the immunohistochemistry (IHC) images of PVR protein using the tissue atlas and pathology atlas panels in the Human Protein Atlas (HPA, https://www.proteinatlas.org/) (28, 29). The associated between *PVR* expression and patient overall survival (OS) was evaluated in TCGA pan-cancer and displayed using R package “forestplot”. To identify the somatic mutations of the patients in TCGA-BLCA cohort, mutation data were downloaded and visualized using the “maftools” package in R (30). Horizontal histogram showed the genes have the higher mutation frequency in patients with bladder cancer.

### Immune Infiltration Analysis in Bladder Cancer

The stroma and immune scores were measured by Estimation of Stromal and Immune cells in Malignant Tumor tissues using Expression data (ESTIMATE) analysis using R package “estimate”. Tumor purity was calculated according to the algorithm.

The abundance of six immune cells including B cells, CD4+ T cell, CD8+ T cell, neutrophil, macrophage, and dendritic cell (DC) was calculated by Tumor Immune Estimation Resource (TIMER; http://timer.cistrome.org/) algorithm (31). The role of copy number alternations (CNAs) of PVR on immune cell infiltration was also evaluated using this algorithm. Meanwhile, CIBERSORT algorithm was employed for evaluating the percentage of 22 human hematopoietic cell phenotypes, including seven T cell types, naïve and memory B cells, plasma cells, NK cells, and myeloid subsets. Furthermore, Spearman correlation analysis was performed to assess the correlations between *PVR* and immune checkpoints, including but not limited to *PD-L1*, *TIM-3* and *CTLA4*. The results were displayed as heatmaps using R package “pheatmap”.

### RNA Sequencing of Bladder Cancer Samples

We included 6 patients (average age 61.32 ± 10.65 years old) with MIBC diagnosed at the Department of Urology, Xiangya Hospital from June 2016 to September 2020. Patients with the presence of distant metastasis or with other cancers were excluded. The fresh bladder cancer samples were collected and then immediately stored in liquid nitrogen. Total RNA was extracted from the tissues using TRIzol (Invitrogen, Carlsbad, CA, USA) according to the instructions. Subsequently, NanoDrop and Agilent 2100 bioanalyzer (Thermo Fisher Scientific, MA, USA) were used to quantify total RNA. The mRNA library was then constructed. Total RNA was purified and fragmented into small pieces. Then, first- and second-strand cDNA were synthesized. The cDNA fragments were further amplified by polymerase chain reaction (PCR) after incubating with A-tailing mix and RNA Adapter Index for end repair. The qualified double-stranded PCR products were then used to construct the final library (single-stranded circular DNA). Eventually, the 57 qualified bladder cancer samples, named the Xiangya cohort, were further sequenced on a BGISEQ-500 platform (BGI-Shenzhen, China). The gene expression levels were calculated using RSEM (v1.2.12). These detailed procedures have been described in our previous study (32).

### Construction and Validation of Risk Signature Based on PVR-Related Genes

Linkedomics website (http://www.linkedomics.org/) was first implemented to mining *PVR*-related genes (with the | Spearman’s R | > 0.4 and Benjamini-Hochberg false discovery rate (BH-FDR) < 0.05) (33), which were inputted into the Metascape website (http://metascape.org/) for functional and pathway enrichment analysis (34), which involved Gene Ontology (GO) analysis and Kyoto Encyclopedia of Genes and Genomes (KEGG) pathway. Then univariate Cox regression analysis was implemented to filtrate the prognostic *PVR*-related genes. Thereafter, using the R package “glmnet” to conduct least absolute shrinkage and selection operator (LASSO) Cox regression (with the penalty parameter estimated by 10-fold cross-validation), we developed a *PVR*-related gene prognostic signature for the BLCA patients involving 6 *PVR*-related genes. The risk score calculating formula is:

$$Risk\;score=\sum_{i=1}^{n}Coef_{i}\times x_{i}$$

Where *n* = 6, *Coef_i_* means the LASSO coefficients, *x_i_* is the log2(TPM + 1) value of each model genes.

Risk scores were computed for all patients including in our study. For all cohorts, the patients were divided into high risk and low risk groups according to the median risk score. Then risk plots, scatter diagrams, receiver operating characteristic curve (ROC) curves, and Kaplan-Meier survival curves were plotted using the R package “ggplot2”. Differentially expressed genes (DEGs) between two groups were then identified based on the standards of | log2(Fold change) | > 1 and p < 0.05 using the R package “limma” (35), and then used for Gene Set Enrichment Analysis (GSEA) analysis to investigate the hallmarks and KEGG pathways that were more common in the high-risk subgroup compared with the low-risk subgroup.

### Construction of Nomogram Signature

Univariate and multivariate Cox regression analysis was performed to identify the proper variables to build the nomogram. A nomogram was developed based on the results of multivariate Cox proportional hazards analysis to predict the 3- and 5-year overall survival. It provided a graphical representation of the factors, which can be used to calculate the risk of death for an individual bladder cancer patient by the points associated with each risk factor through R package ‘rms’.

### Statistical Analyses

Kaplan-Meier curves and the log-rank test were used to compare the OS between various subgroups, comprising the low- and high-risk subgroups and additional subgroups based on the expression of each of the six *PVR*-related genes. The Student’s *t*-test was used to compare the risk scores between pairs of subgroups based on the following clinicopathologic features: age at initial pathologic diagnosis (< 65 vs. ≥ 65 years old), gender (female vs. male), WHO grade (low vs. high) and pathologic stage (I, II vs. III, IV). Univariate and multivariate Cox regression analyses were utilized to evaluate the independent prognostic value of the risk signature regarding OS.

We performed multivariate Cox regression to establish a nomogram, the calibration plots showed the prognostic predictive accuracy of the nomogram and the C-index was calculated for the nomogram models in both two data sets. These analyses were performed using the R package “rms”. The prognostic ability of the nomogram and other predictors (risk score, age, and pathologic stage) for 3- and 5-year OS was evaluated by decision curve analysis (DCA) curves (R package “rmda”).

Wilcoxon test and Kruskal-Wallis test were used for comparison between 2 groups, and for comparison among > 2 groups, respectively. Spearman correlation analysis was used to gauge the degree of correlation between certain variables, with the following R/rho values being used to judge the strength of correlation: 0 - 0.19, ‘very weak’; 0.20 - 0.39, ‘weak’; 0.40 - 0.59, ‘moderate’; 0.60 - 0.79, ‘strong’; 0.80 - 1.00, ‘very strong’. P < 0.05 was the significance threshold in most analysis. The statistical analysis carried out in this study was using R language (version 4.0.2).

## Results

### *PVR* Expression Is Overexpressed in Bladder Cancer

We obtained data from publicly available databases (TCGA BLCA, n = 408; GSE13507, n = 165; GSE32894, n = 224) to evaluate the mRNA expression levels of *PVR* in bladder cancer. TCGA BLCA cohort was divided into TCGA285 and TCGA123 for training and internal validation, respectively. The characteristics of patients in the training and validation cohorts were summarized in **Table 1**.

**Table 1:** Characteristics of patients in training cohort and validation cohorts from open databases.

Characteristics	Training cohort	Internal validation cohort	External validation cohort 1	External validation cohort 2
	TCGA285	TCGA123	GSE13507	GSE32894
	n = 285	n = 123	n = 165	n = 224
**Age (y)**				
< 65	102	48	69	70
>= 65	183	75	96	154
NA	0	0	0	0
**Gender**				
Female	79	28	30	61
Male	206	95	135	163
NA	0	0	0	0
**Grade**				
Low	15	6	105	129
High	268	116	60	93
NA	2	1	0	2
**Stage**				
0	0	0	23	0
I	2	0	80	0
II	90	40	26	0
III	101	39	23	0
IV	91	43	13	0
NA	1	1	0	224*

*No detailed pathologic stage data reported in GSE32894 cohort.

**Figure 1:**
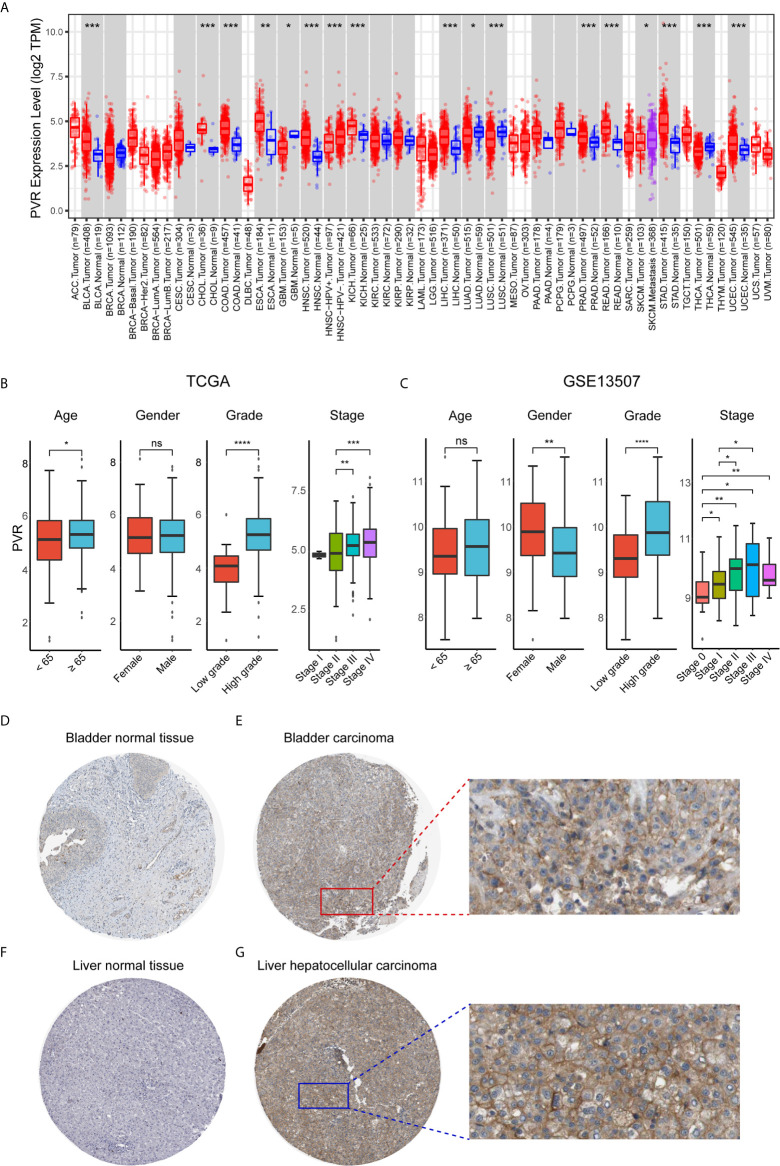
*PVR* expression profiles in human normal and cancer tissues. **(A)** *PVR* expression levels in different tumor types from TCGA database were analyzed by TIMER2.0 (*P < 0.05, **P < 0.01, ***P < 0.001). **(B, C)** The *PVR* expression of different subgroups for age, gender, WHO grade, pathologic stage in TCGA and GSE13507 cohorts. **(D–G)** Representative IHC images of PVR protein expression in bladder normal tissues, bladder carcinoma tissues, liver normal tissues, and hepatocellular carcinoma tissues, respectively. ****P < .0001. ns, not significant.

First, we evaluated *PVR* expression levels in various common cancer types including bladder cancer, and found that tumor samples demonstrated significantly up-regulated *PVR* expression compared to normal bladder tissues (**Figure 1A**), suggesting an association with bladder cancer development. Subsequent stratification analysis demonstrated higher *PVR* expression in higher WHO grade and higher stage in two data sets (**Figures 1B, C**), indicating the potential role of PVR in governing the malignancy properties of bladder cancer. In the TCGA cohort, elders had relatively higher *PVR* expression levels, while *PVR* was significantly increased in female patients in GEO cohort. Such difference was partially caused by the inter-heterogeneity of two cohorts. Immunohistochemistry showed PVR protein was mainly distributed in cytoplasm and membrane, and was moderately expressed in hepatocellular carcinoma and urothelial carcinoma compared to corresponding normal tissues respectively (**Figures 1D–G**). The detailed information of IHC results were summarized in **Supplementary Table 2**.

### *PVR* Expression Predicts Poorer Survival Probability in Bladder Cancer

**Figure 2:**
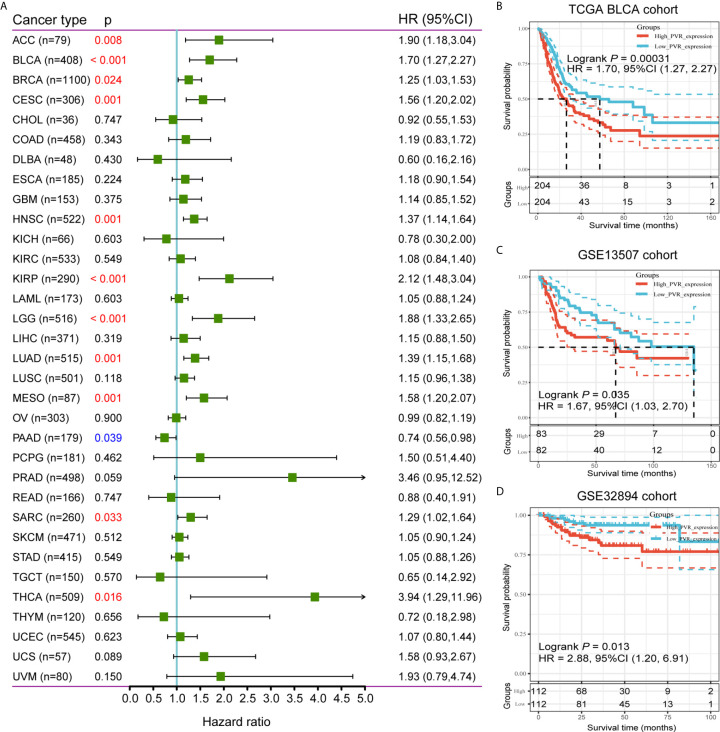
Survival analysis comparing *PVR* ^high^ and *PVR* ^low^ groups in cancers. **(A)** Relationship between *PVR* expression and patient overall survival across 33 cancer types in TCGA database. **(B–D)** Kaplan-Meier survival curves based on *PVR* expression in TCGA BLCA, GSE13507, and GSE32894 cohorts.

To further investigate the prognostic potential of *PVR*, we used TCGA RNA-seq and clinical data to analyze the prognosis across 33 TCGA cancer types. As shown in **Figure 2A**, elevated *PVR* expression was significantly related to a poorer overall survival in adrenocortical carcinoma (ACC; HR = 1.90, 95% CI = 1.18-3.04, p = 0.008), BLCA (HR = 1.70, 95% CI = 1.27-2.27, p < 0.001), breast invasive carcinoma (BRCA; HR = 1.25, 95% CI = 1.03-1.53, p = 0.024), cervical squamous cell carcinoma (CESC; HR = 1.56, 95% CI = 1.20-2.02, p = 0.001), head and neck squamous cell carcinoma (HNSC; HR = 1.37, 95% CI = 1.14-1.64, p = 0.001), kidney renal papillary cell carcinoma (KIRP; HR = 2.12, 95% CI = 1.48-3.04, p < 0.001), brain lower grade glioma (LGG; HR = 1.88, 95% CI = 1.33-2.65, p < 0.001), lung adenocarcinoma (LUAD; HR = 1.39, 95% CI = 1.15-1.68, p = 0.001), mesothelioma (MESO; HR = 1.58, 95% CI = 1.20-2.07, p = 0.001), sarcoma (SARC; HR = 1.29, 95% CI = 1.02-1.64, p = 0.033), and thyroid carcinoma (THCA; HR = 3.94, 95% CI = 1.29-11.96, p = 0.016). On the contrary, increased *PVR* expression was associated with the better prognosis in pancreatic adenocarcinoma (PAAD; HR = 0.74, 95% CI = 0.56-0.98, p = 0.039). Based on the median values of *PVR* expression in bladder cancers, samples were divided in to *PVR* ^low^ and *PVR* ^high^ subgroups for subsequent Kaplan-Meier survival analysis. *PVR* ^high^ patients exhibited significantly shorter overall survival time in TCGA BLCA (HR = 1.70, 95% CI = 1.27-2.27, p < 0.001), GSE13507 (HR = 1.67, 95% CI = 1.03-2.70, p = 0.035), and GSE32894 cohorts (HR = 2.88, 95% CI = 1.20-6.91, p = 0.013) (**Figures 2B–D**). Furthermore, we conducted survival analysis in stratified subgroups, and found that high *PVR* mRNA expression was correlated with poorer OS in several subgroups, including gender—male, race—white, mutation burden—low, and patients with enriched CD4+ memory T cells, CD8+ T cells, eosinophils, as well as mesenchymal stem cells (**Supplementary Table 3**). These results reveal that *PVR* might serve as a predictor of prognosis in bladder cancer patients.

### Mutational Analysis Reveals Distinct Genomic Alterations of *PVR* in BLCA

Based on *PVR* expression levels, the somatic mutation profiles revealed that mutations in *TP53* (47%), *TTN* (45%), *KMT2D* (29%), *MUC16* (27%), *KDM6A* (26%), *ARID1A* (24%), *PIK3CA* (21%) were significantly enriched in BLCA samples (**Supplementary Figure 1A**). The mutation rate of *PVR* was 0.97%, and 4 missense mutation sites were detected locating between amino acids 0 and 417 of PVR protein (**Supplementary Figure 1B**). Meanwhile, the distribution of variants according to variant classification, variant type, and single nucleotide variants (SNV) class was displayed as cohort summary plot (**Supplementary Figure 1C**). Among all the genomic alterations, missense mutation was the predominate type, with C > T and C > G standing as the most common SNV classes.

Samples were further classified into four groups according to the somatic copy number alterations (SCNA) profile of *PVR*, and the distribution of infiltrating immune cells among the four categories was compared in TIMER webtool. The arm-level deletion and arm-level gain of *PVR* significantly impacted the infiltration level of CD4+ T cells, while the high amplification of *PVR* was significantly correlated with elevated abundance of macrophages (**Supplementary Figure 1D**). Incidentally, further correlation analysis suggested that the *PVR* expression was poorly associated with tumor mutational burden (TMB) and microsatellite instability (MSI) across pan-cancer types in TCGA database (**Supplementary Figures 1E–F**), suggesting that PVR was rather unlikely to affect tumorigenesis by participating in the process of genetic alterations.

### PVR Is Involved in Immune Processes and Tumor Microenvironment

**Figure 3:**
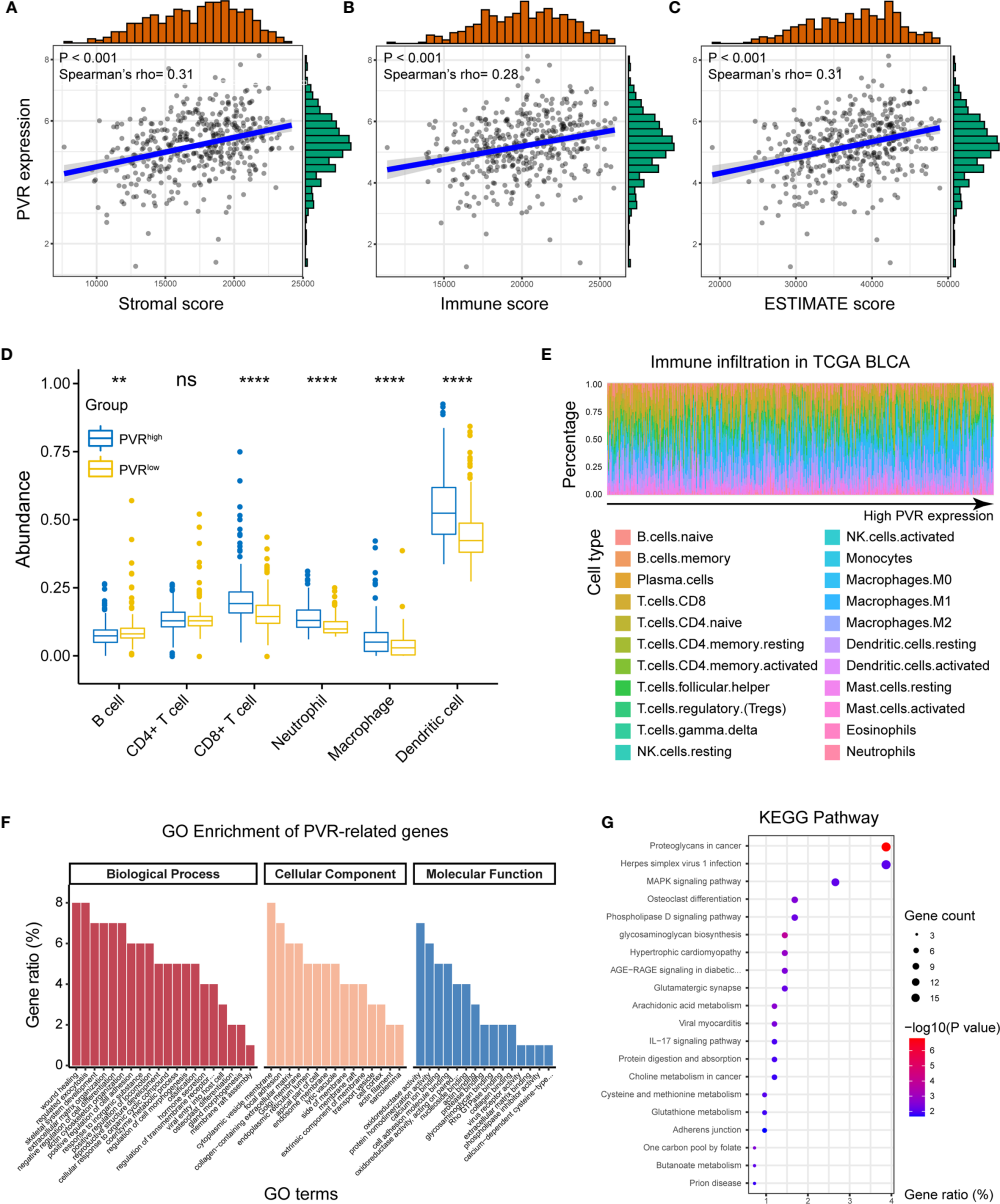
PVR correlating with tumor purity, immune infiltration, and immune-oncologic pathways. **(A–C)** *PVR* expression correlates with stromal, immune, and ESTIMATE scores, respectively in TCGA BLCA cohort. **(D)** The abundance of 6 immune infiltrates between *PVR* ^high^ and *PVR* ^low^ groups in TCGA BLCA cohort (based on TIMER algorithm). **(E)** The abundance of 22 immune infiltrates in TCGA BLCA cohort (based on CIBERSORT algorithm). **(F, G)** GO and KEGG analysis of *PVR*-related genes in bladder cancer. **P < .01, ****P < .0001. ns, not significant.

Positively correlated with stromal, immune, and ESTIMATE scores, *PVR* mRNA expression level demonstrated a significant opposite trend to purity in bladder cancer (**Figures 3A–C**). Based on TIMER and CIBERSORT algorithm, we got the abundance of several immune infiltrates in TCGA BLCA, GSE13507 and Xiangya cohorts. In TCGA BLCA cohort, the abundance of CD8+ T cell, macrophage, neutrophil, and dendritic cell was significantly increased in *PVR* ^high^ group, while B cell abundance was decreased (**Figure 3D**). The correlations between *PVR* expression and six immune infiltrates based on TIMER webtool were consistent with that (**Supplementary Figure 2A**). Results based on CIBERSORT also suggested that samples with higher *PVR* expression tended to possess lower infiltration levels of B cell, as well as higher infiltration levels of monocyte, macrophage, and DC in TCGA data set (**Figure 3E**). Immune infiltration landscape based on TIMER and CIBERSORT algorithms in GSE13507 was showed in **Supplementary Figures 2B–C**.

**Figure 4:**
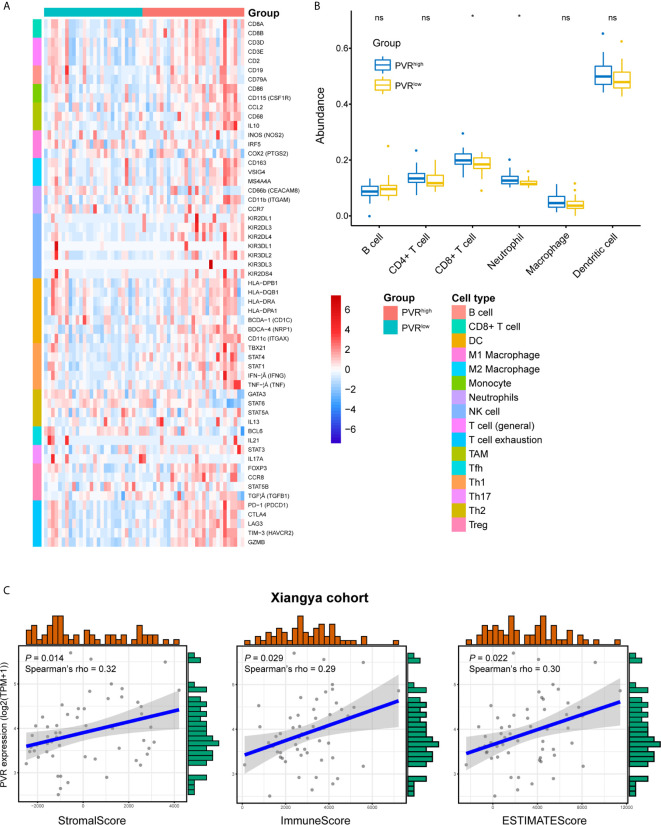
Validation of *PVR*’s role in tumor purity and immune infiltration using Xiangya cohort. **(A)** The heatmap of markers on multiple immune infiltrates. (DC, dendritic cell; NK cell, natural killer cell; TAM, tumor associated macrophages; Tfh, follicular helper T cell; Th1, type 1 helper T cell; Th17, type 17 helper T cell; Th2, type 2 helper T cell; Treg, regulatory T cell) **(B)** The abundance of 6 immune infiltrates between *PVR* ^high^ and *PVR* ^low^ groups in Xiangya cohort (based on TIMER algorithm). **(C)** *PVR* expression correlates with stromal, immune, and ESTIMATE scores, respectively in Xiangya cohort. *P < .05. ns, not significant.

We next used our Xiangya cohort to investigate whether *PVR* correlates with immune infiltration and tumor purity within bladder cancer. In general, we employed our own cohort and get the result similar to that in TCGA BLCA cohort. *PVR* was positively correlated with immune markers on multiple immune cells, including CD8+ T cells, general T cells, monocytes, tumor associated macrophages (TAM), NK cells, type 1 helper T cells (Th1), regulatory T cells (Tregs), and T cells exhaustion (Tex) in Xiangya cohort (**Figure 4A**). Furthermore, the abundance of CD8+ T cell, macrophage, neutrophil, and dendritic cell was increased in *PVR* ^high^ group, while B cell abundance was decreased in our cohort. But significance was only observed in immune subsets of CD8+ T cell and neutrophil (**Figure 4B**). In consistent with TCGA cohort, *PVR* expression was also significantly correlated to tumor purity, as it was positively associated with stromal, immune and ESTIMATE scores (**Figure 4C**).

By the Linkedomics website, 414 *PVR*-related genes (|Spearman R| > 0.4, FDR < 0.001; **Supplementary Table 4**) were identified and used for functional analysis. *PVR*-related genes were mostly located on cytoplasmic vesicle membrane, involved in biological processes including wound healing, regulated exocytosis, extracellular matrix organization, and positive regulation of cell adhesion. In terms of molecular functions, these genes were mainly involved in oxidoreductase activity cell adhesion molecule binding (**Figure 3F**). Meanwhile, KEGG pathway enrichment analysis showed that *PVR*-related genes were closely involved in oncologic and immune processes, including proteoglycans in cancer, Herpes simplex virus 1 (HSV1) infection, and mitogen-activated protein kinase (MAPK) signaling pathway, et al. (**Figure 3G**). And the correlation heatmap showed that *PVR* was significantly correlated with several immune checkpoints including Neuropilin-1 (NRP1), hepatitis A virus cellular receptor 2 (HAVCR2), CD276, programmed cell death 1 ligand 2 (PDCD1LG2), CD274, and CD44 (**Supplementary Figure 2C**).

### Construction of Risk Signature Based on *PVR*-related Genes in the Training Set

**Figure 5:**
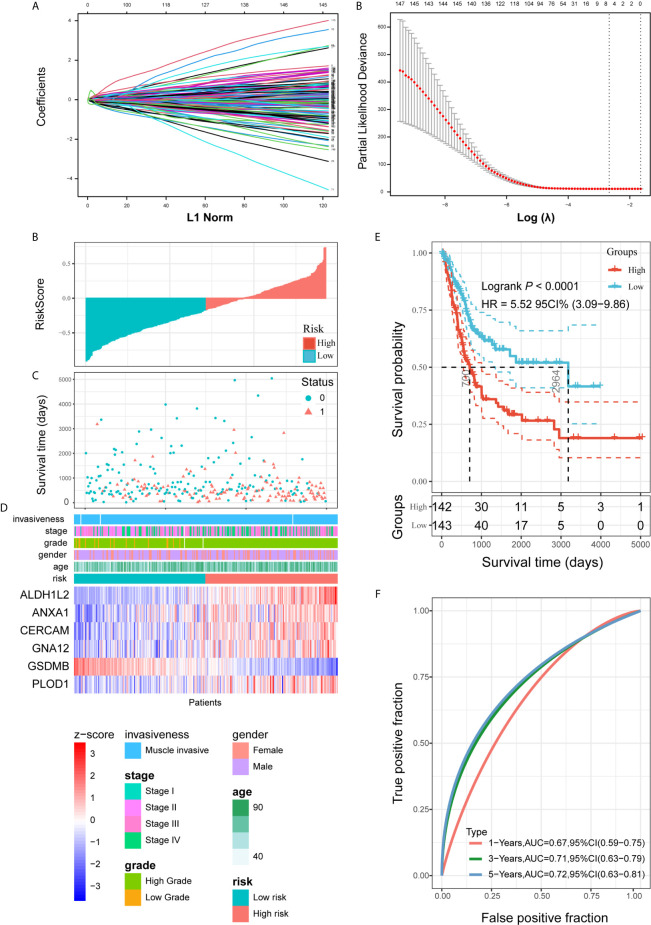
Construction and analysis of risk signature based on *PVR*-related genes in the training set. **(A)** LASSO analysis with eligible lambda value. **(B, C)** Risk score and survival status of each patient in the training cohort. **(D)** Expression pattern of six candidate *PVR*-related genes in the high- and low-risk groups, annotated with corresponding clinicopathologic characteristics. **(E)** Kaplan-Meier analysis of patients in the high- and low-risk groups. **(F)** Time-dependent ROC analysis of risk score in predicting prognoses.

Combined with the prognostic information, univariate Cox regression was then implemented to screen prognostic genes from the 414 *PVR*-related genes in the TCGA data sets. Finally, we found that 148 *PVR*-related genes were significantly correlated with the OS in the training set (p < 0.05). To build the risk signature based on *PVR*-related genes for forecasting the OS of BLCA patients, we performed a LASSO Cox analysis on the basis of the 148 prognostic genes in the TCGA285 data set and it generated the LASSO risk signature which contained 6 *PVR*-related genes and corresponding coefficients (**Figure 5A**). Six *PVR*-related genes, including *ALDH1L2*, *ANXA1*, *CERCAM*, *GNA12*, *PLOD1*, and *GSDMB*, were included in the risk signature. **Table 2** showed that *GSDMB* was a protective factor with HR < 1, while *ALDH1L2*, *ANXA1*, *CERCAM*, *GNA12*, and *PLOD1* were risk factors (HR > 1) in BLCA patients. The Kaplan-Meier survival curves confirmed that higher expression of *GSDMB* and lower expression of *ALDH1L2*, *ANXA1*, *CERCAM*, *GNA12*, and *PLOD1* were associated with better OS in the training set (**Supplementary Figure 3A–F**).

**Table 2:** Risk signature based on *PVR*-related genes in training set.

Factor	Gene	Description	*P*	HR	95% CI	LASSO coefficient
Risk	*ALDH1L2*	Aldehyde dehydrogenase 1 family, member L2	< 0.0001	1.44	1.24-1.68	0.096626563
	*ANXA1*	Annexin A1	0.0009	1.18	1.07-1.31	0.035594173
	*CERCAM*	Cerebral endothelial cell adhesion molecule	< 0.0001	1.32	1.15-1.52	0.016800805
	*GNA12*	Guanine nucleotide binding protein alpha 12	0.0003	1.51	1.21-1.88	0.006655467
	*PLOD1*	Procollagen-lysine, 2-oxoglutarate 5-dioxygenase 1	0.0004	1.42	1.17-1.72	0.003607814
Protective	*GSDMB*	Gasdermin B	< 0.0001	0.69	0.59-0.80	-0.175524875

Risk scores and survival status distributions were plotted in **Figures 5B, C**. Divided into low- and high-risk subgroups based on the median value of risk scores, the expression of six risk signature genes and the corresponding clinicopathologic features of TCGA285 samples were displayed in **Figure 5D**. It clearly showed that *ALDH1L2*, *ANXA1*, *CERCAM*, *GNA12*, and *PLOD1* expression increased with increasing risk score, whereas the expression of the *GSDMB* decreased with elevating risk score. Their expression levels were also related to the clinicopathologic features of bladder cancer, such as age, WHO grade, and pathologic stage. Kaplan-Meier survival curves depicted that BLCA patients with higher risk scores significantly had worse clinical outcomes in the training set (**Figure 5E**). And the ROC curves demonstrated that the risk signature harbored a promising ability to predict OS in the TCGA285 cohort (1-year AUC = 0.67, 3-year AUC = 0.71, 5-year OS = 0.72; **Figure 5F**).

### Internal and External Validation Demonstrates Stability of the Risk Signature

**Figure 6:**
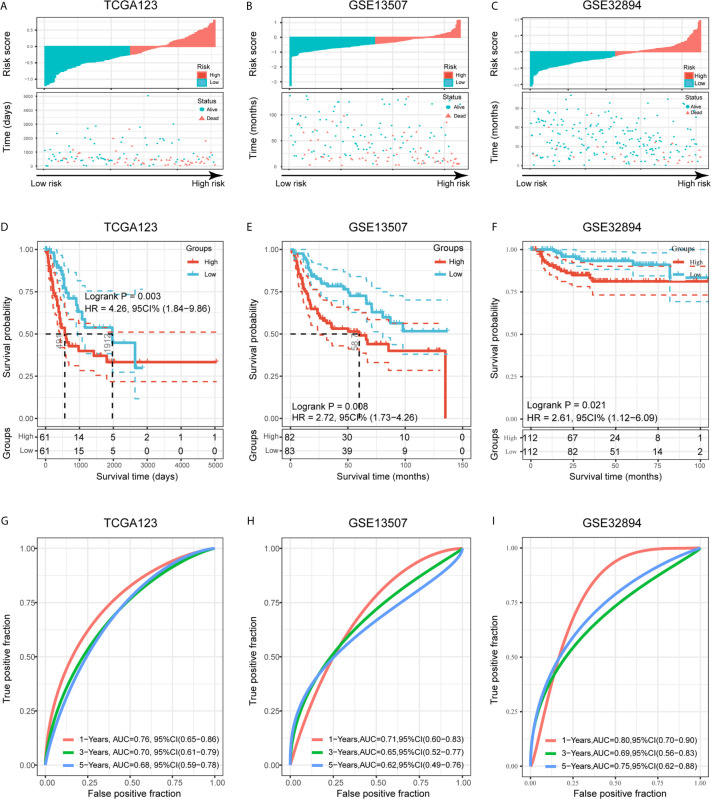
Validation of the risk signature in three validation cohorts. **(A–C)** Risk score of each patient in the three data sets. **(D–F)** Kaplan-Meier analysis of patients in the high- and low-risk subgroups in the three data sets. **(G–I)** Time-dependent ROC analysis of risk score in predicting prognoses in the three data sets.

To validate the prognostic ability of the established risk signature, we calculated risk scores for patients in the TCGA123, GSE13507, and GSE32894 cohorts using the same risk formula, and then assigned them to low- and high-risk groups based on the median risk score (**Figures 6A–C**). BLCA patients with higher risk scores had lower OS rates and a shorter OS time in the validation cohorts (p < 0.05; **Figures 6D–F**). The ROC analysis also indicated that the risk signature had a prognostic value for patients with bladder cancer in the internal validation cohort (TCGA123; 1-year AUC = 0.76, 3-year AUC = 0.70, 5-year AUC = 0.68; **Figure 6G**), external validation cohort 1 (GSE13507; 1-year AUC = 0.71, 3-year AUC = 0.65, 5-year AUC = 0.62; **Figure 6H**), and external validation cohort 2 (GSE32894; 1-year AUC = 0.80, 3-year AUC = 0.69, 5-year AUC = 0.75; **Figure 6I**). These results showed that the risk signature derived from *PVR*-related genes had a robust and stable OS-predictive ability for BLCA patients. Intriguingly, principal component analysis (PCA) based on Gene Expression Profiling Interactive Analysis 2 (GEPIA2; http://gepia2.cancer-pku.cn/) showed that these six signature genes could distinguish bladder cancer from other tumors of urinary system, including kidney chromophobe (KICH), kidney renal clear cell carcinoma (KIRC), KIRP, and prostate adenocarcinoma (PRAD) (**Supplementary Figures 3G–H**) (36).

To better assess the prognostic ability of the risk signature, we performed a stratification analysis to confirm whether it retains its ability to predict OS in various subgroups. In contrast with patients with lower risk, higher risk BLCA patients had worse OS in both patients aged < 65 and ≥ 65 years old (**Supplementary Figures 4A**). Likewise, we confirmed that the risk signature retained its ability to predict OS for male or female patients (**Supplementary Figures 4B**), patients with different tumor grades (**Supplementary Figures 4C**) and patients with different stages (**Supplementary Figures 4D**). These data indicated that it could be a stable predictor for BLCA patients.

### Risk Signature Correlates With Clinicopathologic Characteristics and Immune Microenvironment of BLCA

**Figure 7:**
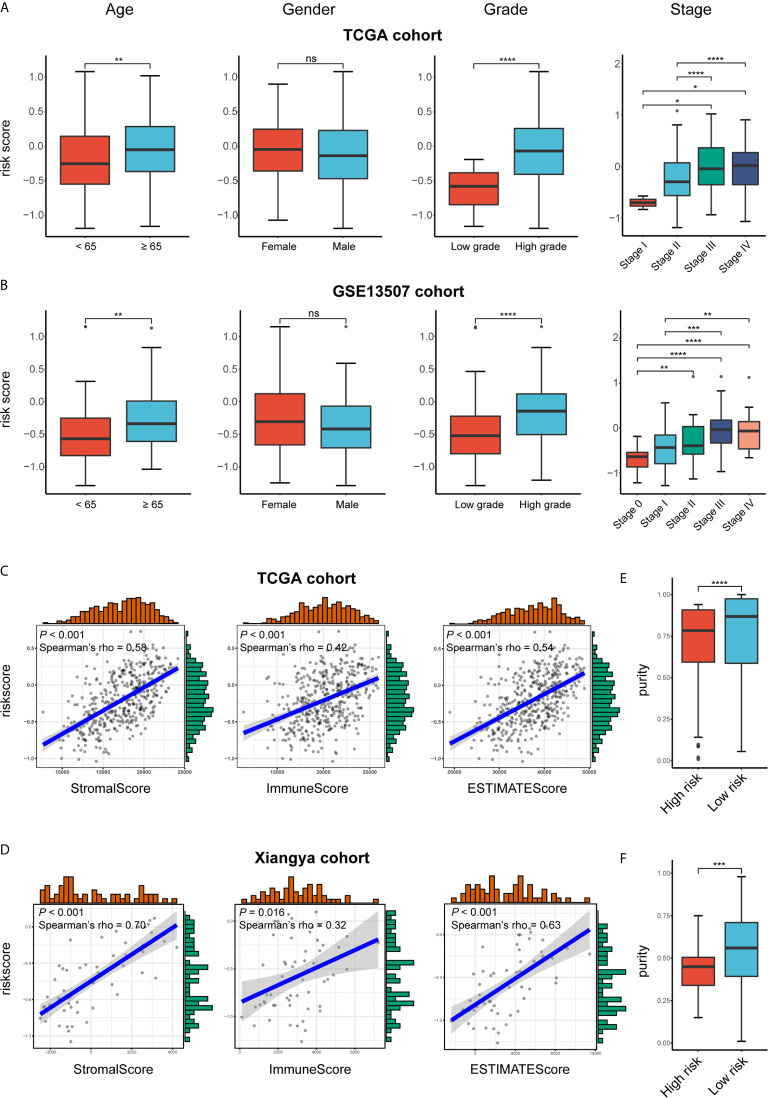
Exploration the relationship between clinicopathologic features, immune purity, and the risk signature. **(A, B)** Risk scores of different subgroups for age, gender, WHO grade, histologic stage in TCGA BLCA and GSE13507 cohorts. **(C, D)** The correlation between stromal, immune, and ESTIMATE scores and risk score in the high- and low-risk groups in TCGA BLCA and GSE13507 cohorts. **(E, F)** Tumor purity between high- and low-risk groups in TCGA BLCA and Xiangya cohorts. *P < .05, **P < .01, ***P < .001, ****P < .0001. ns, not significant.

We attempted to identify whether clinicopathologic features were associated with the risk signature. The results revealed that BLCA patients with increasing age, higher WHO grade, and higher pathologic stage significantly had higher risk scores, while the risk score was not associated with gender (**Figures 7A, B**).

**Figure 8:**
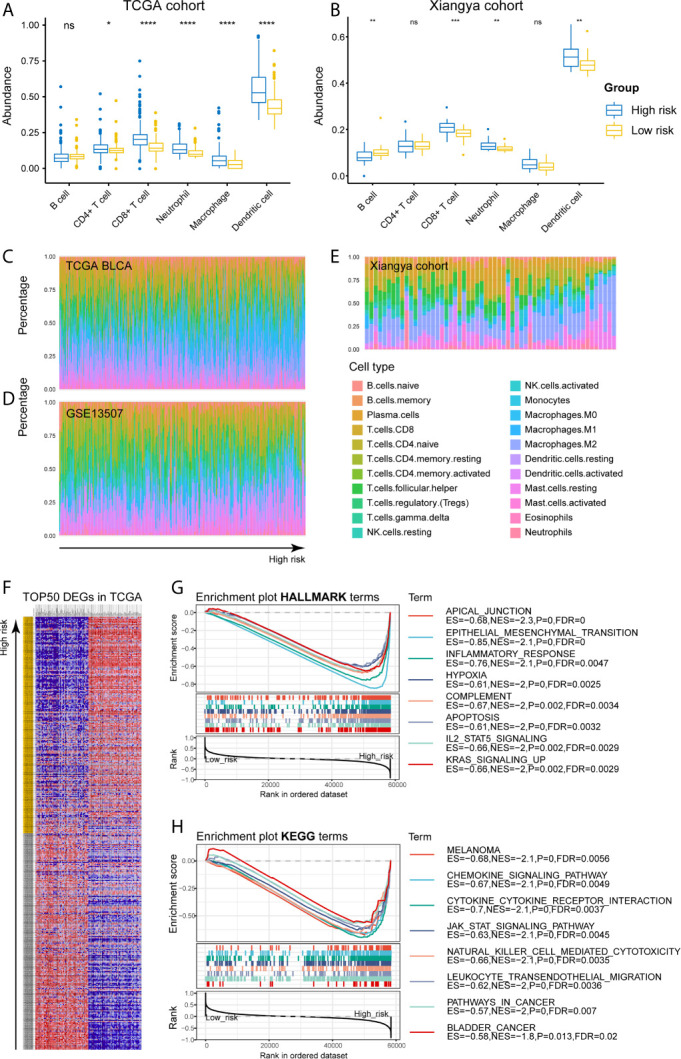
High-risk samples sharing higher abundance of immune infiltrates, tumor hallmarks, and immune-oncologic terms. **(A, B)** The abundance of 6 immune infiltrates between high- and low-risk groups in TCGA BLCA and Xiangya cohorts (based on TIMER algorithm). **(C–E)** The abundance of 22 immune infiltrates in TCGA BLCA, GSE13507 and Xiangya cohorts (based on CIBERSORT algorithm). **(F)** TOP 50 positive and negative DEGs between high- and low-risk groups in bladder cancer. **(G, H)** Enriched HALLMARK and KEGG terms for high-risk gene set. *P < .05, **P < .01, ***P < .001, ****P < .0001. ns, not significant.

As for the immune microenvironment of bladder cancer, the stroma, immune, and ESTIMATE scores were strongly and positively correlated with risk scores, whereas the tumor purity was lower in the high-risk group compared with the low-risk one in both TCGA and Xiangya cohorts (p < 0.05) (**Figures 7C–F**). Moreover, samples with high risk demonstrated higher abundance of CD8+ T cell, neutrophil, macrophage, and dendritic cell in those two cohorts (p < 0.05; **Figures 8A, B**). Meanwhile, CIBERSORT results revealed consistent results that samples with higher risk scores tended to harbor higher abundance of monocytes, macrophages, and dendritic cells in TCGA, GSE13507 and Xiangya cohorts (**Figures 8C–E**). The validation of the correlation between risk signature and tumor purity as well as immune infiltration was displayed in **Supplementary Figures 3I–K**, which is consistent with that in both TCGA and Xiangya cohorts.

These results confirmed the establishment of the risk signature and its association with the prognosis and immune microenvironment of bladder cancers.

For investigating the potential biological processes and pathways involving in the molecular heterogeneity between the low- and high-risk subgroups, we identified 2571 differential expression genes (DEGs; | log2 (fold change) | > 1, p < 0.05) between the subgroups in the TCGA cohort. TOP 50 positive and negative DEGs in BLCA were exhibited in **Figure 8F**. GSEA revealed that several tumor hallmarks were enriched in the samples with high risk, such as epithelial-mesenchymal transition, inflammatory response, hypoxia, complement, IL2-STAT5 signaling, KRAS signaling (FDR < 0.05; **Figure 8G**) and so on. And several oncological KEGG terms were enriched in high-risk group, including melanoma, chemokine signaling pathway, cytokine-cytokine receptor interaction, JAK-STAT signaling pathway, NK cell mediated cytotoxicity, leukocyte transendothelial migration, pathways in cancers, and bladder cancer (FDR < 0.05; **Figure 8H**). These results shed lights on the cellular biological effects related to the identified risk signature.

The association between risk stratification and therapeutic efficacy was also explored. In the TCGA cohort, patients in the high-risk group who received chemoradiotherapy had longer survival compared with those receiving other therapies (p = 0.0066; **Supplementary Figure 4H**). Chemotherapy exhibited a potent efficacy in high-risk group in both TCGA BLCA (p = 0.0035; **Supplementary Figure 4J**) and GSE13057 (p = 0.016; **Supplementary Figure 4L**) cohorts. On the contrary, chemo- and radiotherapy had no significant effect on improving the outcome of patients in the low-risk group (**Supplementary Figure 4E–G, K**). Besides, the risk score was significantly correlated with the expression of recognized immune checkpoints, especially NRP1, PDCD1LG2, CD86, and CD44 in the TCGA cohort (Spearman’ rho > 0.5, P < 0.001) (**Supplementary Figure 5A**). Therefore, the risk stratification was associated with the efficacy of chemotherapy and might predict the efficacy of immunotherapy in bladder cancers.

### Construction and Validation of the Risk Signature-Based Nomogram

We used univariate and multivariate Cox analyses to assess whether the established risk signature was an independent prognostic factor for patients with BLCA. Based on the data of BLCA samples in the TCGA data set, univariate Cox analysis indicated that age at initial diagnosis, pathologic stage, *PVR* expression level, and risk score were remarkably associated with OS (p < 0.05). Subsequent multivariate Cox analysis further showed that age, stage, and risk score were independent predictors of OS (p < 0.05) (**Table 3**). The conclusion was validated in the GSE13507 data set, which confirmed age, stage, and risk signature as independent predictors of OS for BLCA patients (**Table 4**). These results indicated that our risk signature, as an independent prognostic indicator, might be useful for clinical prognosis evaluation.

**Table 3:** Univariate and multivariate Cox analyses in TCGA BLCA cohort.

Factor	TCGA								
	Univariate					Multivariate			
	HR	lower 95%CI	upper 95%CI	p		HR	lower 95%CI	upper 95%CI	p
**Age**	1.03	1.02	1.05	< 0.001		1.02	1.01	1.04	0.002
**Gender**									
Male vs. female	0.88	0.64	1.22	0.443		0.91	0.65	1.26	0.565
**Grade**									
High vs. low grade	2.90	0.72	11.71	0.136		0.90	0.21	3.81	0.887
**Stage**									
Stage III, IV vs. I, II	2.21	1.53	3.20	< 0.001		1.82	1.24	2.67	0.002
***PVR* expression**	1.33	1.14	1.55	< 0.001		1.00	0.82	1.21	0.979
**Risk score**	4.92	3.08	7.86	< 0.001		3.94	2.26	6.87	< 0.001

**Table 4:** Univariate and multivariate Cox analyses in GSE13507 cohort.

Factor	GSE13507								
	Univariate					Multivariate			
	HR	Lower 95% CI	Upper 95% CI	p		HR	Lower 95% CI	Upper 95% CI	p
**Age**	1.07	1.04	1.10	< 0.001		1.07	1.05	1.10	< 0.001
**Gender**									
Male vs. female	0.64	0.36	1.14	0.129		0.77	0.41	1.45	0.419
**Grade**									
High vs. low grade	2.74	1.69	4.43	< 0.001		0.86	0.47	1.58	0.622
**Stage**									
Stage III, IV vs. I, II	5.47	3.31	9.04	< 0.001		5.26	2.82	9.83	< 0.001
***PVR* expression**	1.54	1.12	2.11	0.007		0.99	0.70	1.40	0.955
**Risk score**	2.72	1.73	4.26	< 0.001		1.64	1.09	2.90	0.008

**Figure 9:**
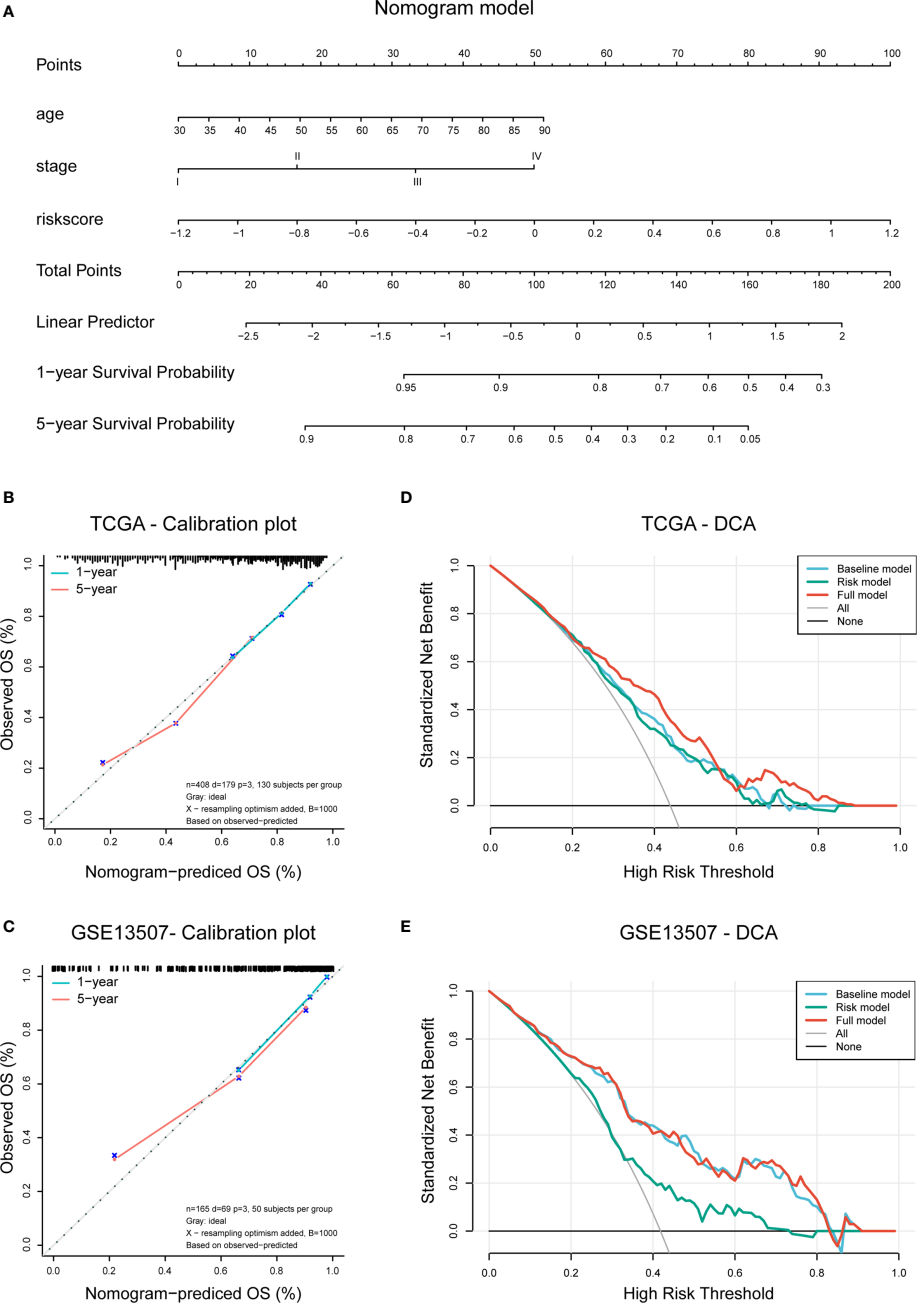
Construction and validation of the nomogram. **(A)** Nomogram based on age, tumor stage, and risk score. **(B, C)** Calibration plots of the nomogram for predicting the probability of OS at 3- and 5-year in the TCGA BLCA and GSE13507 cohorts. **(D, E)** DCA plots evaluating the predictor efficacy of nomogram in the TCGA BLCA and GSE113507 cohorts (Baseline model: age and stage included; Risk model: risk score included; Full model: the nomogram).

To create a clinically applicable quantitative tool to predict the OS of BLCA patients, we constructed a nomogram model including the risk score, age at initial diagnosis, and tumor pathologic stage in the TCGA data set (**Figure 9A**). Calibration plots showed that the observed vs. predicted rates of 3- and 5-year OS revealed perfect concordance in the TCGA BLCA (**Figure 9B**) and GSE13507 cohorts (**Figure 9C**). C-index was also calculated for assessing the predictive ability of the nomogram in both two data sets as and results showed a stable and robust predictive power (C-index for the TCGA data set: 0.807; and GSE13507 data set: 0.705). These data indicated that the nomogram has a robust and stable ability to predictive the OS for BLCA patients. The DCA results indicated that the model combining clinicopathologic characteristics (age and stage) and risk signature owned a better predictive potency than the two models alone (**Figures 8D, E**). The clinical impact curves of two cohorts were displayed in **Supplementary Figures 5B, C**.

## Discussion

Muscle invasive bladder cancer is an aggressive and heterogeneous disease associated with high morbidity and mortality (2). Molecular profiling of bladder cancer has helped to enhance our understanding of tumor biology and identify several therapeutic targets, such as PD−1/PD−L1 axis. Over the past decade, immunotherapies targeting PD-1/PD-L1 axis have demonstrated the ability to promote patient prognosis (3). However, a considerable number of patients still presented poor responsiveness. Therefore, novel immune targets are in urgent need for the hope to promote response rate and improve patient outcomes. We employed comprehensive bioinformatic analyses to elucidate that PVR is definitely a critical molecule and helps clinicians to optimize a novel approach to the management of bladder cancer, especially MIBC. We believe that *PVR* and its related genes are good predictors of outcome in MIBC, and targeting PVR will show unique efficacy in future clinical practice.

In this report, we assessed the profile of *PVR*, and found that *PVR* expression was increased in various cancer types including bladder cancer. Besides, BLCA samples with higher WHO grade or higher pathologic stage tended to express higher level of *PVR*, highly suggesting that *PVR* was associated with the progression and malignancy of bladder cancer. Further analysis showed that increased *PVR* mRNA expression was related to poorer overall survival in multiple cancers including BLCA, re-analysis in two independent GEO cohorts also identified *PVR* as a significant risk factor in bladder cancer. We thus suppose that *PVR* might impact the tumor properties and patient prognosis *via* the intervention on immune infiltration in TME, since it was significantly correlated with tumor purity, recognized immune checkpoints, and immune infiltrates in several independent cohorts including TCGA, GSE13507, and Xiangya cohorts. The hypothesis was collaborated by subsequent enrichment analysis, which found that *PVR*-related genes were involved in several immune processes and oncological pathways, such as wound healing, proteoglycans in cancer, as well as MAPK signaling pathway.

Previous studies have employed bioinformatic tools to detect and validate promising molecular targets in cancers, such as Siglec15 in bladder cancer (32). In our study, a total of 797 BLCA patients from the TCGA and GEO data sets were included to exploit the prognostic significance of risk signature based on *PVR*-related genes. 148 *PVR*-related genes were confirmed to have prognostic value, and 6 of them (*ALDH1L2*, *ANXA1*, *CERCAM*, *GNA12*, *GSDMB*, and *PLOD1*) were used to establish a risk signature for predicting the OS of BLCA patients. Previous study suggested that *ALDH1L2* knockdown could inhibit distant metastasis without significantly affecting the growth of subcutaneous tumors in the melanoma mice models (37). ANXA1 was confirmed to participate in regulatory T cell (Treg)-mediated immune suppression in triple-negative breast cancer and can promote nasopharyngeal carcinoma growth and metastasis (38). Besides, CERCAM is a probable cell adhesion protein involved in leukocyte transmigration and participates in tumorigenesis (39). As for GNA12, it was identified as an important signature involved in pathways in inflammatory bowel diseases and prostate cancer (40, 41). Importantly, PLOD1 is aberrantly expressed and correlated with poorer outcome in bladder cancer (42), which is consistent with our finding. Moreover, a recent study reported that NK cells and cytotoxic T lymphocytes killed GSDMB-positive cells through pyroptosis, a form of proinflammatory cell death executed by the gasdermin family of pore-forming proteins (43). The study establishes gasdermin-mediated pyroptosis as a cytotoxic lymphocyte-killing mechanism, and indicates that lower *GSDMB* expression reflects enhanced antitumor immunity. In summary, the published works suggested that these candidate genes and corresponding proteins were involved in the tumor progression across various cancer types, which were consistent to our risk signature, where *ALDH1L2*, *ANXA1*, *CERCAM*, *GNA12*, and *PLOD1* were risk factors, whereas *GSDMB* served as a protective factor for bladder cancer.

Based on the median risk score, patients with primary bladder cancer were divided into the low- and high-risk subgroups in all included cohorts. The high-risk group demonstrate worse clinical outcomes, higher abundance of immune infiltrates, as well as enrichment of tumor hallmarks (e.g., epithelial-mesenchymal transition, inflammatory response, hypoxia, complement, IL2-STAT5 signaling, and KRAS signaling) and certain malignant terms (e.g., chemokine signaling pathway, cytokine-cytokine receptor interaction, JAK-STAT signaling pathway, NK cell mediated cytotoxicity, leukocyte transendothelial migration, pathways in cancers, and bladder cancer), remarkably indicating that the constructed risk signature could reflect the immune infiltration involving tumor microenvironment and robustly predicted survival in primary bladder cancer. Multivariate Cox regression analysis showed that our risk signature was an independent risk factor for OS. Furthermore, combining the established signature with age and pathologic stage, we constructed a nomogram model, which exhibited a robust ability to predict OS in BLCA patients in both TCGA BLCA and GSE13507 cohorts.

A key finding in our study is that *PVR* expression and the establish risk signature are both positively correlated with the immune infiltrates, especially CD8+ T cells, macrophages, and DCs. Existing evidence has suggested that PVR can inhibit the function of NK cells *via* interacting with TIGIT, thereby suppressing the deteriorating effects against tumor cells. Therefore, we supposed that PVR could mediate similar immune functions in bladder cancer. On the one hand, it acts as a coinhibitory molecule, mediating the immunosuppressive phenotype of NK and T cells. On the other hand, PVR and related molecules may participate in the process of immune adhesion and antigen presentation between malignant cells and lymphocytes by affecting the binding of cytokines-cytokine receptors (44). Obviously, further work will be necessary in order to assess whether PVR exerts such functions in bladder cancer. Meanwhile, emerging evidence showed that therapies targeting PVR/TIGIT/CD96 pathways could enhance anti-tumor capacity across certain cancer types (45, 46). We believe that immunotherapy blocking PVR/TIGIT interaction, allied with current immune checkpoint blockades, will improve the survival outcome based on our findings.

There were several limitations in our study. Firstly, detailed pathologic staging data are not provided in the GSE32894 cohort, so we did not use this cohort for Cox regression analysis or validation of the nomogram. Secondly, the sample size of our own Xiangya cohort was too small, thus the accuracy of the results is open to question. Thirdly, clinical data such as tumor stage and survival time were not available in Xiangya cohort. Although we employ these samples to validate our conclusion through public databases that *PVR* is associated with multiple immune markers and strongly correlated with tumor purity and immune infiltration, we still need multicenter cohorts with large sample size and complete clinical data to verify our conclusions.

In a nutshell, we employed data from three independent cohorts in open database, and our own cohort to explore the expression and mutation profile of *PVR* (*CD155*) in bladder cancer. Involved in multiple immune processes and oncological terms, *PVR* impacts immune infiltration and correlates with poorer outcome in patients with primary bladder cancer. A risk signature and subsequent nomogram are established based on *PVR*-related genes to robustly predict patient overall survival, with a potential for clinical utilization.

## Data Availability Statement

The data analyzed in this study is subject to the following licenses/restrictions: the Xiangya cohort used in the article belongs to the Department of Urology, Xiangya Hospital, Central South University. The cohort is still in expansion and is not permitted to make public at this time point. Requests to access these datasets should be directed to hujiao1993@163.com.

## Ethics Statement

Written informed consent was obtained from the individual(s) for the publication of any potentially identifiable images or data included in this article.

## Author Contributions

CL and WY performed the data analysis and interpreted the data. JH and JC collected the samples in our cohort and they were responsible for the subsequent RNA sequencing of them. CL prepared the draft. WY performed the visualization. BO and HL revised the manuscript. JC and XZ designed the research and supervised all the work. All authors contributed to the article and approved the submitted version.

## Funding

This work was supported by the National Natural Science Foundation of China (81873626, 81902592), Hunan Natural Science Foundation (2020JJ5884), Hunan Province Key R&D Program (2019SK2202), and Xiangya Hospital Youth Fund (2018Q09).

## Conflict of Interest

The authors declare that the research was conducted in the absence of any commercial or financial relationships that could be construed as a potential conflict of interest.

## References

[1] Antoni S Ferlay J Soerjomataram I Znaor A Jemal A Bray F. Bladder Cancer Incidence and Mortality: A Global Overview and Recent Trends. Eur Urol (2017) 71(1):96–108. 10.1016/j.eururo.2016.06.010 27370177

[2] Kamat AM Hahn NM Efstathiou JA Lerner SP Malmström PU Choi W. Bladder Cancer. Lancet (London Engl) (2016) 388(10061):2796–810. 10.1016/s0140-6736(16)30512-8 27345655

[3] Lobo N Mount C Omar K Nair R Thurairaja R Khan MS. Landmarks in the Treatment of Muscle-Invasive Bladder Cancer. Nat Rev Urol (2017) 14(9):565–74. 10.1038/nrurol.2017.82 28675174

[4] Mitra AP Cote RJ. Molecular Screening for Bladder Cancer: Progress and Potential. Nat Rev Urol (2010) 7(1):11–20. 10.1038/nrurol.2009.236 20062071

[5] Lenis AT Lec PM Chamie K Mshs MD. Bladder Cancer: A Review. Jama (2020) 324(19):1980–91. 10.1001/jama.2020.17598 33201207

[6] Kamat AM Bellmunt J Galsky MD Konety BR Lamm DL Langham D. Society for Immunotherapy of Cancer Consensus Statement on Immunotherapy for the Treatment of Bladder Carcinoma. J Immunother Cancer (2017) 5(1):68. 10.1186/s40425-017-0271-0 28807024 PMC5557323

[7] Hirota T Irie K Okamoto R Ikeda W Takai Y. Transcriptional Activation of the Mouse Necl-5/Tage4/PVR/CD155 Gene by Fibroblast Growth Factor or Oncogenic Ras Through the Raf-MEK-ERK-AP-1 Pathway. Oncogene (2005) 24(13):2229–35. 10.1038/sj.onc.1208409 15688018

[8] Okumura G Iguchi-Manaka A Murata R Yamashita-Kanemaru Y Shibuya A Shibuya K. Tumor-Derived Soluble CD155 Inhibits DNAM-1-mediated Antitumor Activity of Natural Killer Cells. J Exp Med (2020) 217(4):1. 10.1084/jem.20191290 PMC7144518 32040157

[9] O’Donnell JS Madore J Li XY Smyth MJ. Tumor Intrinsic and Extrinsic Immune Functions of CD155. Semin Cancer Biol (2020) 65:189–96. 10.1016/j.semcancer.2019.11.013 31883911

[10] Yu X Harden K Gonzalez LC Francesco M Chiang E Irving B. The Surface Protein TIGIT Suppresses T Cell Activation by Promoting the Generation of Mature Immunoregulatory Dendritic Cells. Nat Immunol (2009) 10(1):48–57. 10.1038/ni.1674 19011627

[11] Deuss FA Watson GM Fu Z Rossjohn J Berry R. Structural Basis for CD96 Immune Receptor Recognition of Nectin-like Protein-5, Cd155. Struct (London Engl: 1993) (2019) 27(2):219–28. 10.1016/j.str.2018.10.023 30528596

[12] Zhuo B Li Y Gu F Li Z Sun Q Shi Y. Overexpression of CD155 Relates to Metastasis and Invasion in Osteosarcoma. Oncol Lett (2018) 15(5):7312–8. 10.3892/ol.2018.8228 PMC5920504 29725446

[13] Braun M Aguilera AR Sundarrajan A Corvino D Stannard K Krumeich S. CD155 on Tumor Cells Drives Resistance to Immunotherapy by Inducing the Degradation of the Activating Receptor CD226 in CD8(+) T Cells. Immunity (2020) 53(4):805–23. 10.1016/j.immuni.2020.09.010 33053330

[14] Chauvin JM Ka M Pagliano O Menna C Ding Q DeBlasio R. Il15 Stimulation With TIGIT Blockade Reverses CD155-Mediated NK-Cell Dysfunction in Melanoma. Clin Cancer Res: an Off J Am Assoc Cancer Res (2020) 26(20):5520–33. 10.1158/1078-0432.Ccr-20-0575 PMC8045409 32591463

[15] Nakai R Maniwa Y Tanaka Y Nishio W Yoshimura M Okita Y. Overexpression of Necl-5 Correlates With Unfavorable Prognosis in Patients With Lung Adenocarcinoma. Cancer Sci (2010) 101(5):1326–30. 10.1111/j.1349-7006.2010.01530.x PMC11158505 20331633

[16] Carlsten M Norell H Bryceson YT Poschke I Schedvins K Ljunggren HG. Primary Human Tumor Cells Expressing CD155 Impair Tumor Targeting by Down-Regulating DNAM-1 on NK Cells. J Immunol (Baltimore Md: 1950) (2009) 183(8):4921–30. 10.4049/jimmunol.0901226 19801517

[17] Pende D Spaggiari GM Marcenaro S Martini S Rivera P Capobianco A. Analysis of the Receptor-Ligand Interactions in the Natural Killer-Mediated Lysis of Freshly Isolated Myeloid or Lymphoblastic Leukemias: Evidence for the Involvement of the Poliovirus Receptor (CD155) and Nectin-2 (Cd112). Blood (2005) 105(5):2066–73. 10.1182/blood-2004-09-3548 15536144

[18] Gromeier M Lachmann S Rosenfeld MR Gutin PH Wimmer E. Intergeneric Poliovirus Recombinants for the Treatment of Malignant Glioma. Proc Natl Acad Sci USA (2000) 97(12):6803–8. 10.1073/pnas.97.12.6803 PMC18745 10841575

[19] Nishiwada S Sho M Yasuda S Shimada K Yamato I Akahori T. Clinical Significance of CD155 Expression in Human Pancreatic Cancer. Anticancer Res (2015) 35(4):2287–97. 25862891

[20] Masson D Jarry A Baury B Blanchardie P Laboisse C Lustenberger P. Overexpression of the CD155 Gene in Human Colorectal Carcinoma. Gut (2001) 49(2):236–40. 10.1136/gut.49.2.236 PMC1728395 11454801

[21] Zhang C Wang Y Xun X Wang S Xiang X Hu S. Tigit Can Exert Immunosuppressive Effects on CD8+ T Cells by the CD155/TIGIT Signaling Pathway for Hepatocellular Carcinoma In Vitro. J Immunother (Hagerstown Md: 1997) (2020) 43(8):236–43. 10.1097/cji.0000000000000330 PMC7566309 32804915

[22] Zhang J Zhu Y Wang Q Kong Y Sheng H Guo J. Poliovirus Receptor CD155 is Up-Regulated in Muscle-Invasive Bladder Cancer and Predicts Poor Prognosis. Urol Oncol (2020) 38(2):41. 10.1016/j.urolonc.2019.07.006 31383549

[23] Huang DW Huang M Lin XS Huang Q. CD155 Expression and its Correlation With Clinicopathologic Characteristics, Angiogenesis, and Prognosis in Human Cholangiocarcinoma. OncoTargets Ther (2017) 10:3817–25. 10.2147/ott.S141476 PMC5546808 28814880

[24] Cancer Genome Atlas Research Network. Comprehensive Molecular Characterization of Urothelial Bladder Carcinoma. Nature (2014) 507(7492):315–22. 10.1038/nature12965 PMC3962515 24476821

[25] Kim WJ Kim EJ Kim SK Kim YJ Ha YS Jeong P. Predictive Value of Progression-Related Gene Classifier in Primary non-Muscle Invasive Bladder Cancer. Mol Cancer (2010) 9:3. 10.1186/1476-4598-9-3 20059769 PMC2821358

[26] Lee JS Leem SH Lee SY Kim SC Park ES Kim SB. Expression Signature of E2F1 and its Associated Genes Predict Superficial to Invasive Progression of Bladder Tumors. J Clin Oncol: Off J Am Soc Clin Oncol (2010) 28(16):2660–7. 10.1200/jco.2009.25.0977 20421545

[27] Sjödahl G Lauss M Lövgren K Chebil G Gudjonsson S Veerla S. A Molecular Taxonomy for Urothelial Carcinoma. Clin Cancer Res: an Off J Am Assoc Cancer Res (2012) 18(12):3377–86. 10.1158/1078-0432.Ccr-12-0077-t 22553347

[28] Uhlén M Fagerberg L Hallström BM Lindskog C Oksvold P Mardinoglu A. Proteomics. Tissue-based Map of the Human Proteome. Sci (New York NY) (2015) 347(6220):1260419. 10.1126/science.1260419 25613900

[29] Uhlen M Zhang C Lee S Sjöstedt E Fagerberg L Bidkhori G. A Pathology Atlas of the Human Cancer Transcriptome. Sci (New York NY) (2017) 357(6352):660. 10.1126/science.aan2507 28818916

[30] Mayakonda A Lin DC Assenov Y Plass C Koeffler HP. Maftools: Efficient and Comprehensive Analysis of Somatic Variants in Cancer. Genome Res (2018) 28(11):1747–56. 10.1101/gr.239244.118 PMC6211645 30341162

[31] Li T Fan J Wang B Traugh N Chen Q Liu JS. Timer: A Web Server for Comprehensive Analysis of Tumor-Infiltrating Immune Cells. Cancer Res (2017) 77(21):e108–e10. 10.1158/0008-5472.Can-17-0307 PMC6042652 29092952

[32] Hu J Yu A Othmane B Qiu D Li H Li C. Siglec15 Shapes a non-Inflamed Tumor Microenvironment and Predicts the Molecular Subtype in Bladder Cancer. Theranostics (2021) 11(7):3089–108. 10.7150/thno.53649 PMC7847675 33537076

[33] Vasaikar SV Straub P Wang J Zhang B. LinkedOmics: Analyzing Multi-Omics Data Within and Across 32 Cancer Types. Nucleic Acids Res (2018) 46(D1):D956–63. 10.1093/nar/gkx1090 PMC5753188 29136207

[34] Zhou Y Zhou B Pache L Chang M Khodabakhshi AH Tanaseichuk O. Metascape Provides a Biologist-Oriented Resource for the Analysis of Systems-Level Datasets. Nat Commun (2019) 10(1):1523. 10.1038/s41467-019-09234-6 30944313 PMC6447622

[35] Ritchie ME Phipson B Wu D Hu Y Law CW Shi W. Limma Powers Differential Expression Analyses for RNA-Sequencing and Microarray Studies. Nucleic Acids Res (2015) 43(7):e47. 10.1093/nar/gkv007 25605792 PMC4402510

[36] Tang Z Kang B Li C Chen T Zhang Z. GEPIA2: An Enhanced Web Server for Large-Scale Expression Profiling and Interactive Analysis. Nucleic Acids Res (2019) 47(W1):W556–w60. 10.1093/nar/gkz430 PMC6602440 31114875

[37] Piskounova E Agathocleous M Murphy MM Hu Z Huddlestun SE Zhao Z. Oxidative Stress Inhibits Distant Metastasis by Human Melanoma Cells. Nature (2015) 527(7577):186–91. 10.1038/nature15726 PMC4644103 26466563

[38] Bai F Zhang P Fu Y Chen H Zhang M Huang Q. Targeting ANXA1 Abrogates Treg-mediated Immune Suppression in Triple-Negative Breast Cancer. J Immunother Cancer (2020) 8(1):e000169. 10.1136/jitc-2019-000169 32300050 PMC7204868

[39] Starzyk RM Rosenow C Frye J Leismann M Rodzinski E Putney S. Cerebral Cell Adhesion Molecule: A Novel Leukocyte Adhesion Determinant on Blood-Brain Barrier Capillary Endothelium. J Infect Dis (2000) 181(1):181–7. 10.1086/315163 10608765

[40] Lees CW Barrett JC Parkes M Satsangi J. New IBD Genetics: Common Pathways With Other Diseases. Gut (2011) 60(12):1739–53. 10.1136/gut.2009.199679 21300624

[41] Udayappan UK Casey PJ. C-Jun Contributes to Transcriptional Control of GNA12 Expression in Prostate Cancer Cells. Mol (Basel Switzerland) (2017) 22(4):612. 10.3390/molecules22040612 PMC6153990 28394299

[42] Yamada Y Kato M Arai T Sanada H Uchida A Misono S. Aberrantly Expressed PLOD1 Promotes Cancer Aggressiveness in Bladder Cancer: A Potential Prognostic Marker and Therapeutic Target. Mol Oncol (2019) 13(9):1898–912. 10.1002/1878-0261.12532 PMC6717764 31199049

[43] Zhou Z He H Wang K Shi X Wang Y Su Y. Granzyme A From Cytotoxic Lymphocytes Cleaves GSDMB to Trigger Pyroptosis in Target Cells. Sci (New York NY) (2020) 368(6494):eaaz7548. 10.1126/science.aaz7548 32299851

[44] Lange R Peng X Wimmer E Lipp M Bernhardt G. The Poliovirus Receptor CD155 Mediates Cell-to-Matrix Contacts by Specifically Binding to Vitronectin. Virology (2001) 285(2):218–27. 10.1006/viro.2001.0943 11437656

[45] Renner S Martins AS Streckel E Braun-Reichhart C Backman M Prehn C. Mild Maternal Hyperglycemia in INS (C93S) Transgenic Pigs Causes Impaired Glucose Tolerance and Metabolic Alterations in Neonatal Offspring. Dis Models Mech (2019) 12(8):dmm039156. 10.1242/dmm.039156 PMC6737953 31308048

[46] Zhang Q Bi J Zheng X Chen Y Wang H Wu W. Blockade of the Checkpoint Receptor TIGIT Prevents NK Cell Exhaustion and Elicits Potent Anti-Tumor Immunity. Nat Immunol (2018) 19(7):723–32. 10.1038/s41590-018-0132-0 29915296

