# Supplementary figures and images for: A Poliovirus Receptor (CD155)-Related Risk Signature Predicts the Prognosis of Bladder Cancer
Source: Front Oncol. 2021 Jun 3;11:660273. doi: 10.3389/fonc.2021.660273 (PMC8210672; doi:10.3389/fonc.2021.660273)

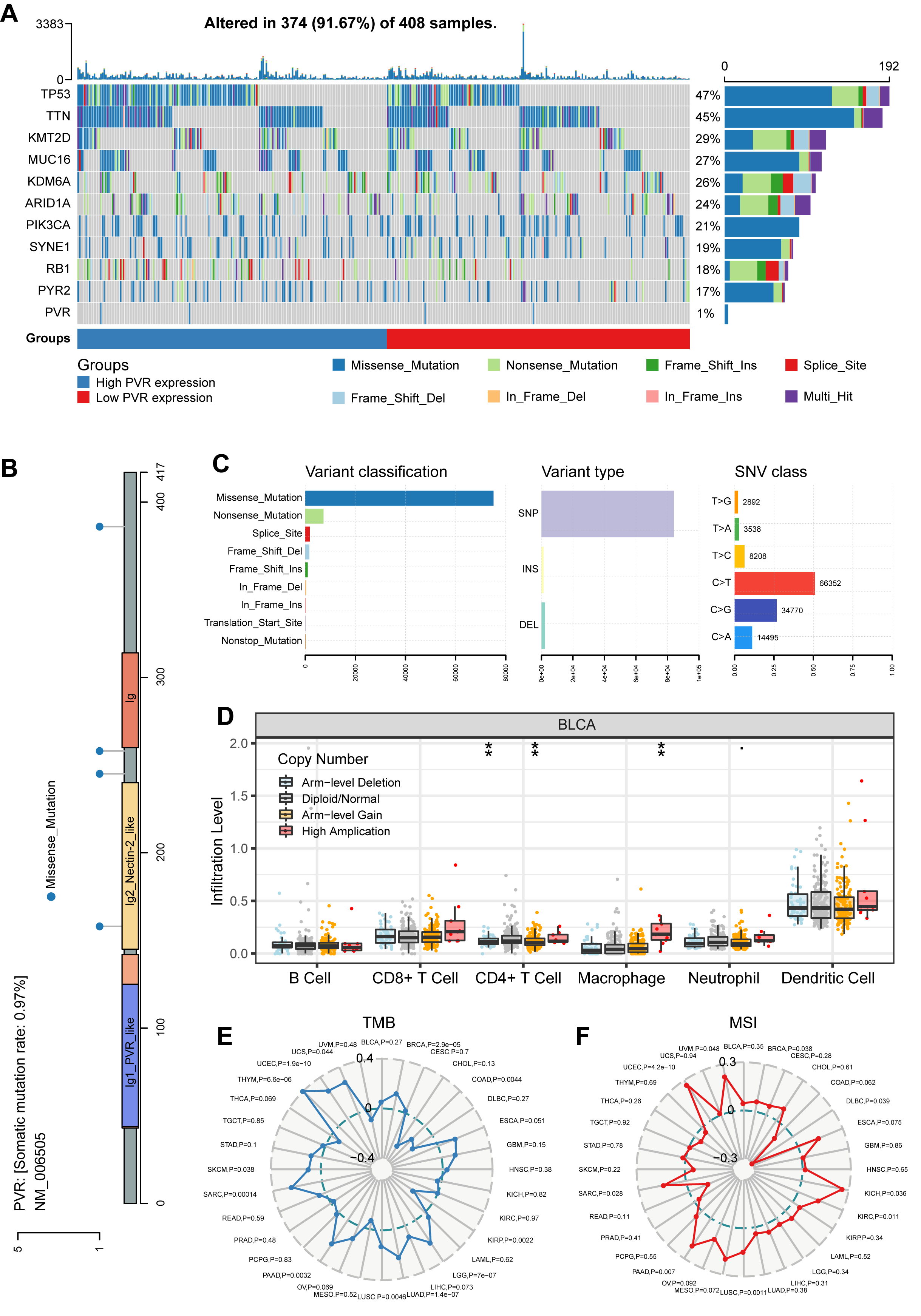

Supplement: Supplementary file 1 [file DataSheet_1.zip › Supplementary files/Supplementary Figure 1. The landscape of PVR mutation in TCGA BLCA cohort.tif]

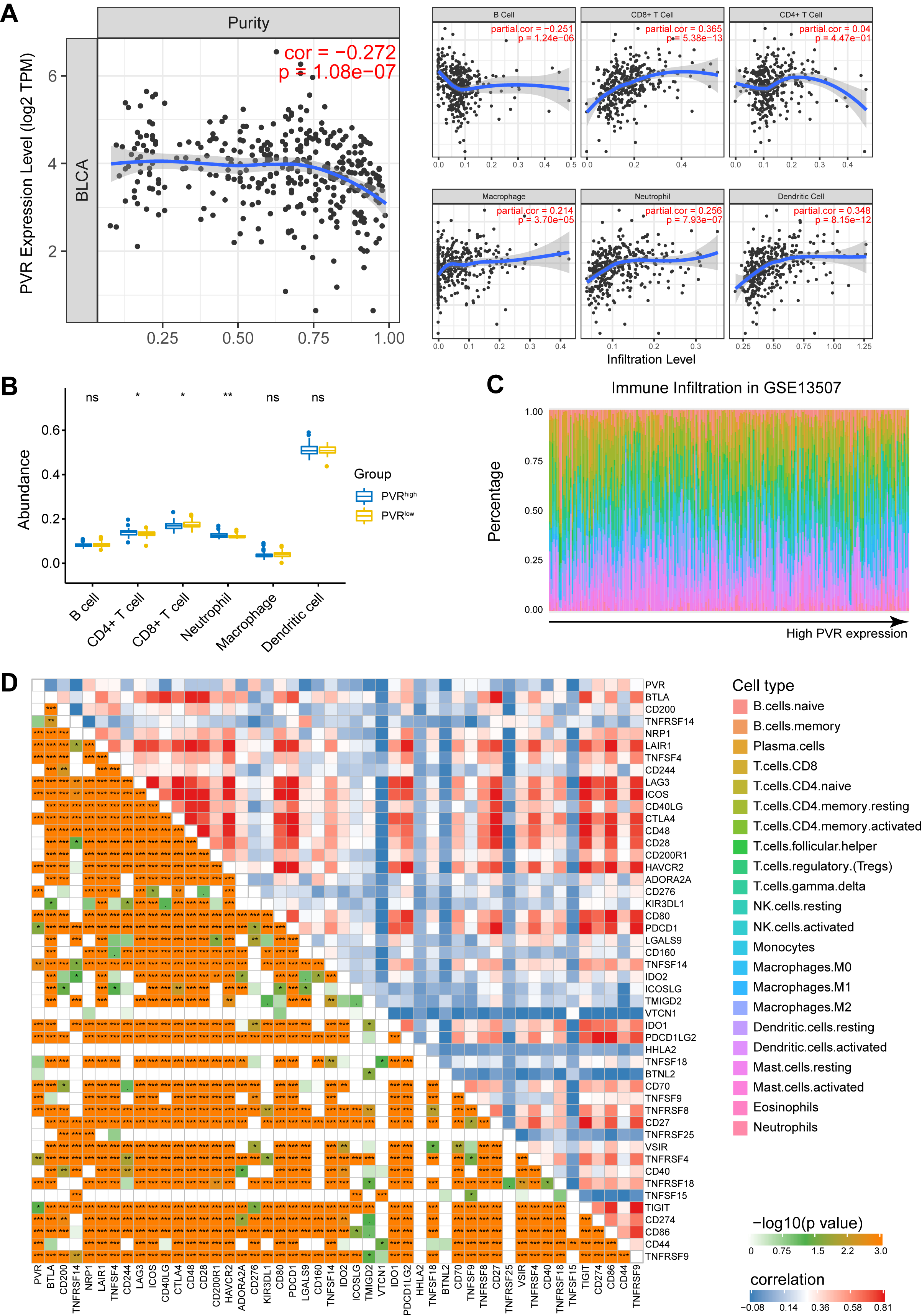

Supplement: Supplementary file 1 [file DataSheet_1.zip › Supplementary files/Supplementary Figure 2. PVR expression correlating with immune infiltration and immune checkpoints.tif]

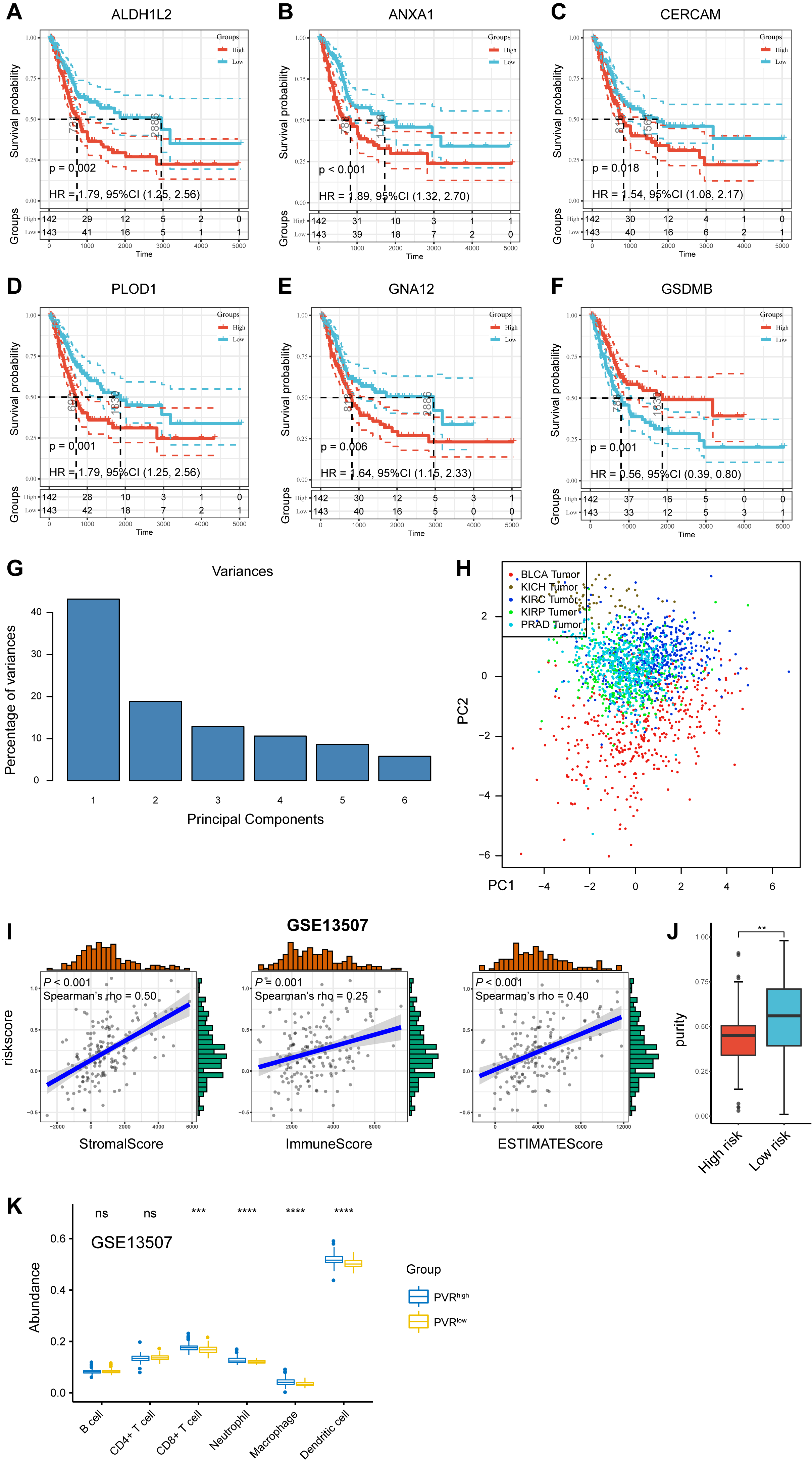

Supplement: Supplementary file 1 [file DataSheet_1.zip › Supplementary files/Supplementary Figure 3. Six candidate risk signature genes and overall survival in training set.tif]

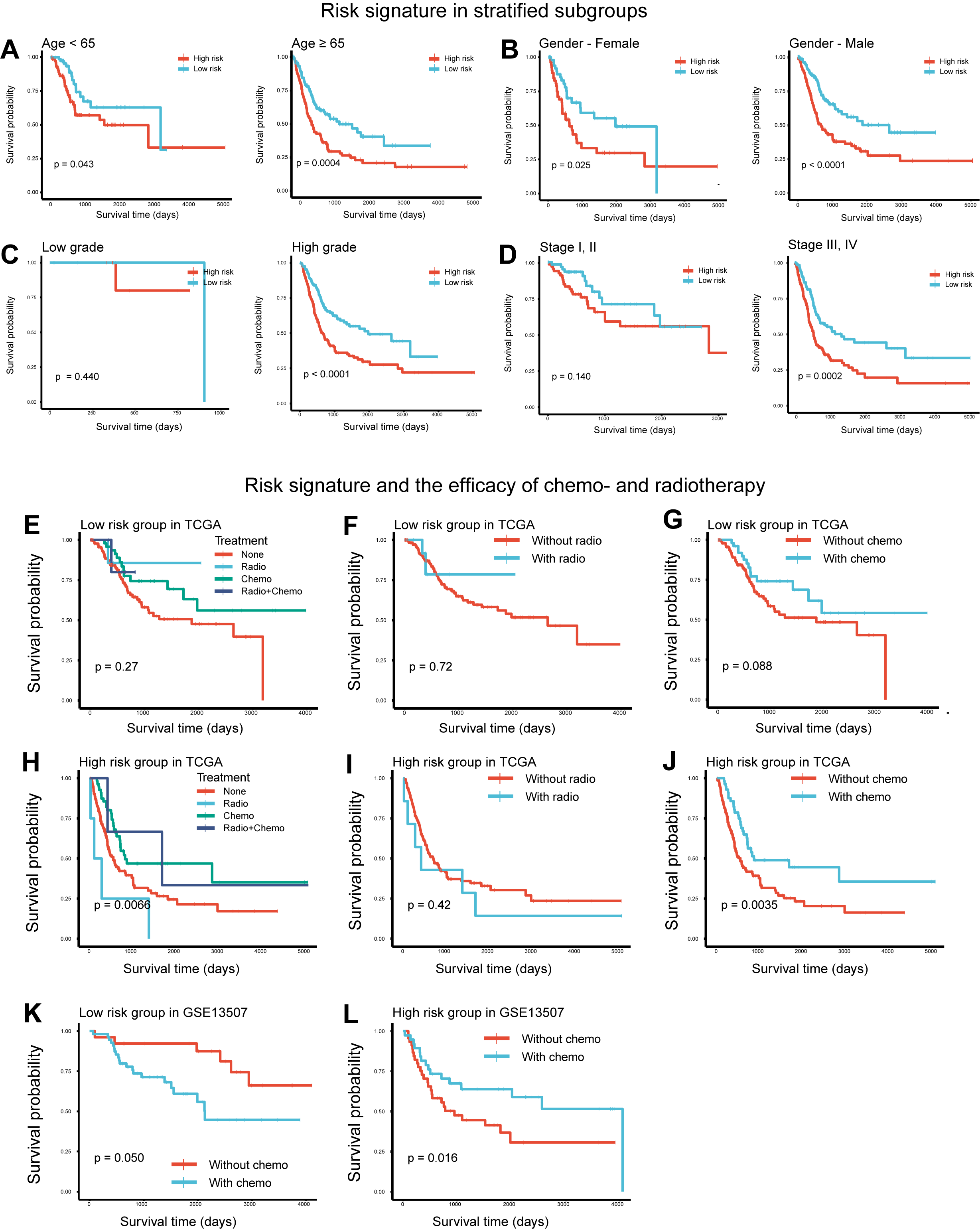

Supplement: Supplementary file 1 [file DataSheet_1.zip › Supplementary files/Supplementary Figure 4. Risk signature in stratified subgroups and predicting treatment efficacy.tif]

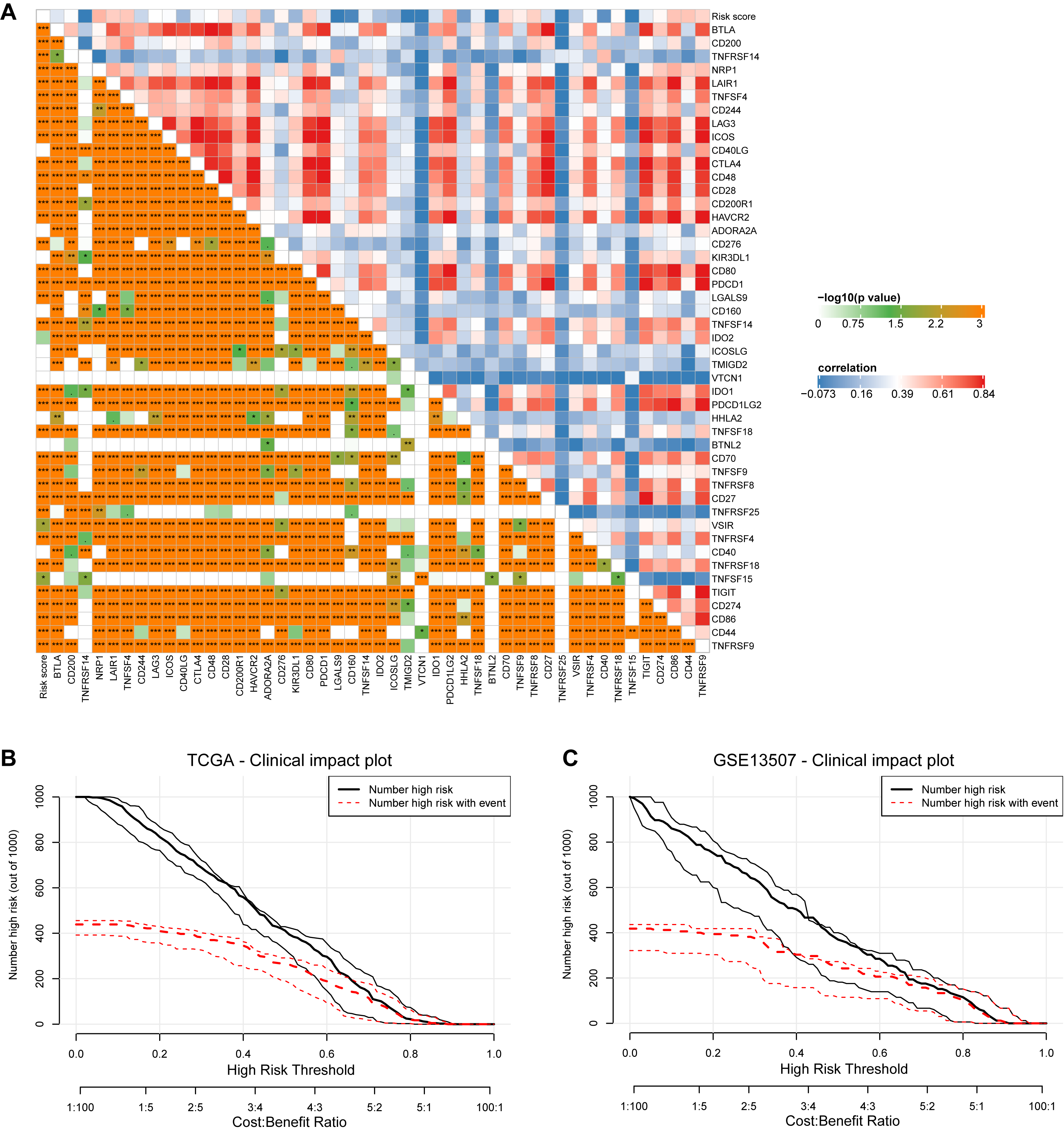

Supplement: Supplementary file 1 [file DataSheet_1.zip › Supplementary files/Supplementary Figure 5. Risk signature and recognized immune chechpoints, and clinical impact plots of the nomogram.tif]
